# Expression of nm23 in the spectrum of pre-invasive, invasive and metastatic breast lesions

**DOI:** 10.1186/1746-1596-3-23

**Published:** 2008-05-30

**Authors:** Amanjit Bal, Kusum Joshi, Rajesh Logasundaram, BD Radotra, Rajinder Singh

**Affiliations:** 1 Department of Histopathology, Post Graduate Institute of Medical Education and Research, Chandigarh, India; 2 Department of General Surgery, Post Graduate Institute of Medical Education and Research, Chandigarh, India

## Abstract

**Background:**

Nm23 protein is a metastasis suppressor protein, expressed in all tissues. Reduced Nm23 expression is related to a high incidence of lymph node and distant metastasis and poor prognosis in patients with cancers. The present study was done to analyze the expression of Nm23 using immunohistochemistry in non-neoplastic and neoplastic breast lesions.

**Methods:**

Sections from 93 samples were studied and classified into non-proliferative breast lesion (13), fibrodenoma (7), proliferative breast lesion (13), carcinoma in situ (20), invasive carcinoma (23) and metastatic deposits in lymph nodes (17).

**Results:**

Nm23 expression in these groups showed a progressive down regulation with increasing neoplastic transformation. On comparing the various groups, nm23 expression was significantly different between the various subgroups with greatest expression in non-proliferative lesions and least in metastatic deposits (p < 0.050).

**Conclusion:**

It is concluded that the modulation of nm23 in a spectrum of breast lesions can be indicative of metastatic phenotype and help to predict the aggressiveness of disease.

## Introduction

Breast cancer is the most common cancer in women of the developed countries with a persistently rising incidence [1]. Extensive research on diagnostic and therapeutic aspects has contributed little to decrease the mortality. This may be attributed to a relative lack of understanding of the natural history of disease and heterogeneity of pre invasive lesions [2].

Various biological makers known to be indicators of prognosis in breast cancer include growth factor receptors, oestrogen receptors, p53, bcl2, Her-2/neu oncogenes and proliferation indices like Ki67. But the most important prognostic factor remains the axillary lymph node status [3,4]. Majority of breast cancer patients succumb to metastatic disease. The molecular basis of the metastatic disease is not known, but activation or inactivation of multiple genes is involved in the various steps of tumor progression [5].

Non metastatic (nm23) gene is the one possible candidate that suppresses the metastatic phenotype [6]. It was first identified by Steeg et al [7] in murine melanoma cells and inverse relationship between metastatic potential and nm23 RNA protein was found in four different metastatic models. Nm23 gene has been localized at chromosome 17q21 and two isoforms (nm23-H1 and nm23-H2) have been identified. Each isoform encodes 17 KD protein having non-specific nucleoside diphosphate kinase (NDPK) activity [8]. Reduced expression of nm23 in breast, hepatocellular and ovarian carcinoma correlates with increased metastatic potential [9-11]. On the contrary, in prostate and lung carcinomas, disease progression is associated with increased nm23 gene expression [12,13].

Breast carcinogenesis is known to be a multistep process and emerges through hyperplasia to atypical hyperplasia and carcinoma in situ [14,15]. Loss of heterozygosity at chromosome 16 and 17 has been observed in ductal carcinoma in situ (DCIS) and lobular carcinoma in situ (LCIS) showing that these genetic abnormalities take place before invasion[16].

The study has been designed to evaluate the expression of nm23/NDPK in breast epithelial lesions that are presumed to represent steps in progression of breast carcinoma.

## Materials and methods

Formalin fixed paraffin-embedded sections of breast lesions were retrieved from the archives of the Department of Histopathology, PGIMER, Chandigarh. There were 93 samples in total; which were classified by the criteria of Dupont and Page[17,18] into six groups as: Non proliferative breast disease (fibrocystic disease, apocrine change and mild epithelial hyperplasia)-13 cases, fibroadenoma-7 cases, proliferative breast disease (moderate and florid hyperplasia)-13 cases, atypical hyperplasia and carcinoma in situ (ductal and lobular)-20 cases, invasive carcinoma(ductal and lobular)-23 cases, and metastatic deposits in lymph nodes-17 cases. None of these cases had received prior chemotherapy.

For immunohistochemistry avidin-biotin method was used. The antibody used was rabbit anti-human monoclonal antibody nm23 at 1:40 dilution (DAKO).

### Quantification of immunostaining

The expression of the antigen was evaluated in a semiquantitative manner as previously described by Bankfalvi et al [19]. Sections were scored based on two parameters: (a) Percentage of positively stained cells; no staining-0, <5% cells stained-1, 5–75% cells stained-2, >75% cells stained-3 (b) Intensity of staining; absent -0 mild-1, moderate-2, strong-3.

Multiplying the values of both parameters generated immunoreactivity scores and this ranged from 0 to 9.

### Statistical analysis

ANOVA test was applied to compare scores between the groups.

## Results

The results are summarized in table 1.

**Table 1:** Nm23 expression in spectrum of benign and malignant breast lesions

**S.No**	**Group**	**No. of cases**	**Mean score (Range)**	**SD**	**COV**	**SE of mean**
1	Non-proliferative lesions	13	7.150(4–9)	2.192	30.649	0.608
2	Fibroadenoma	7	7.860(4–9)	2.034	25.905	0.769
3.	Proliferative lesions	13	5.461(2–9)	1.664	30.469	0.461
4	Carcinoma in situ	20	4.104(2–6)	1.651	40.271	0.369
5.	Invasive carcinoma	23	2.871(0–6)	1.516	52.859	0.316
6.	Lymph node metastasis	17	1.241(0–6)	1.855	150.170	0.449

SD-Standard deviation, COV-coefficient of variation, SE-Standard error

### Nm23 expression in non-proliferative breast lesions

**Figure 1:**
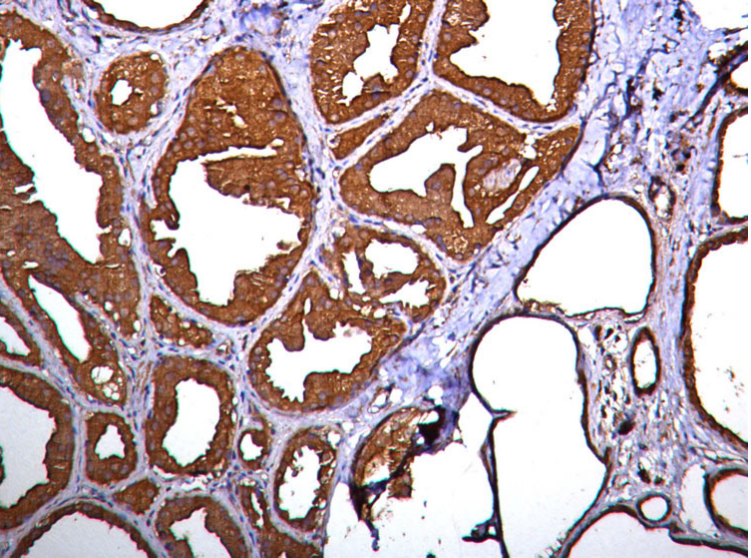
Photomicrograph showing strong nm23 expression in epithelial cells in fibrocystic change (Nm23 immunostain).

This group includes fibrocystic disease, apocrine change and mild epithelial hyperplasia (13 cases). Nm23 was strongly expressed in epithelial as well as stromal cells. Homogeneous staining pattern was observed in cytoplasm of both epithelial and myoepithelial cells (Fig. 1). Immunoreactivity score ranged from 4–9 (mean = 7.150, SD = 2.190). Scores were statistically significanty higher when compared with other groups (proliferative lesions p < 0.050, carcinoma in situ p < 0.001, invasive carcinoma p < 0.001 and lymph node metastasis p < 0.001).

### Nm23 expression in fibroadenoma

**Figure 2:**
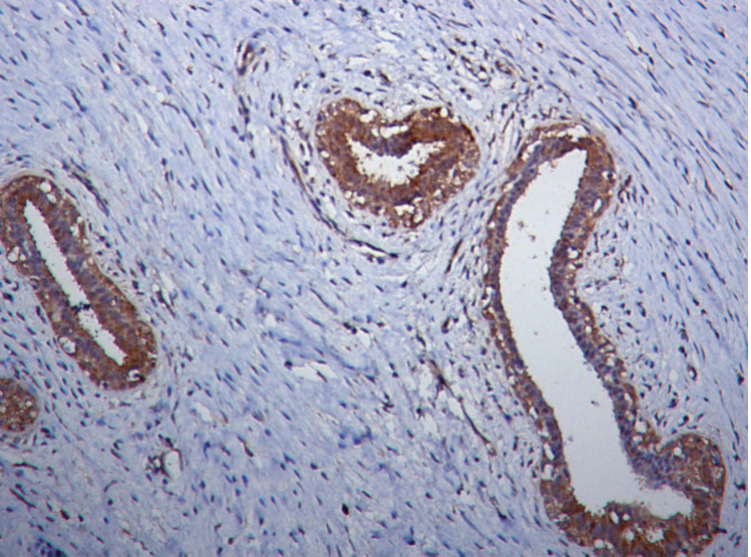
Photomicrograph showing strong nm23 expression in epithelial cells and a few stromal cells in fibroadenoma (Nm23 immunostain).

Seven cases of fibroadenoma were studied. A homogeneous and strong nm23 expression approximating non-proliferative lesions was observed in epithelial and stromal cells (Fig. 2). Score ranged from 4–9 (mean = 7.860, SD = 2.030). Immunoreactivity scores were compared with other groups and the difference was statistically significant (proliferative lesions p < 0.002, carcinoma in situ p < 0.001, invasive carcinoma p < 0.001 and lymph node metastasis p < 0.001). No significant difference was found between fibroadenoma and non-proliferative lesions.

### Nm23 expression in proliferative breast lesions

**Figure 3:**
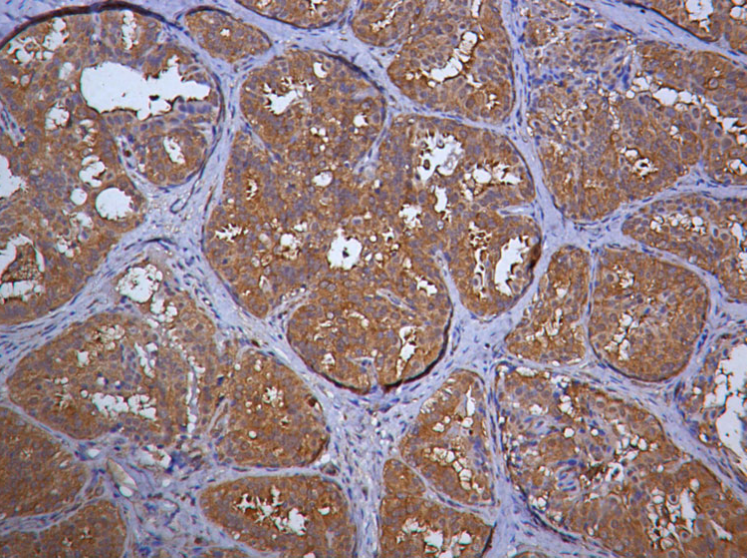
Photomicrograph showing moderate nm23 expression in florid epithelial hyperplasia (Nm23 immunostain).

This group includes moderate and florid hyperplasia(13 cases). Homogeneous moderate staining for nm23 was noted in the hyperplastic areas with a score range of 2–9(mean = 5.460, SD = 1.660) (Fig. 3). Scores varied significantly from scores of carcinoma in situ (p < 0.005), invasive carcinoma(p < 0.001) and lymph node metastasis (p < 0.001).

### Nm23 expression in carcinoma in situ

Twenty cases of carcinoma in situ including one case of lobular carcinoma in situ showing areas of atypical ductal hyperplasia were studied. Weak to moderate immunoreactivity was noted in epithelial cells with score ranging from 2–6(mean = 4.100, SD = 1.650). Carcinoma in situ had greater nm23 expression than invasive carcinoma(p < 0.002) and lymph node metastasis (p < 0.001). No significant difference was observed in staining intensity of comedo and non-comedo carcinoma in situ.

### Nm23 expression in invasive carcinoma

**Figure 4:**
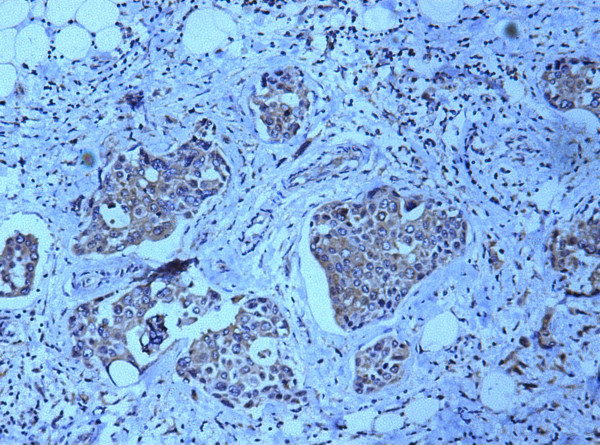
Photomicrograph showing weak to absent nm23 expression in invasive ductal carcinoma cells (Nm23 immunostain).

This group includes 23 cases of invasive carcinoma. Most of these showed extremely reduced nm23 expression in tumour cells with score range of 0–6(mean = 2.870, SD = 1.510)(Fig. 4). The scores varied significantly when compared with lymph node meatastasis (p < 0.001). Periductal elastosis was strongly stained. There was no difference in staining intensity between ductal and lobular subtypes.

### Nm23 expression in lymph node metastasis

**Figure 5:**
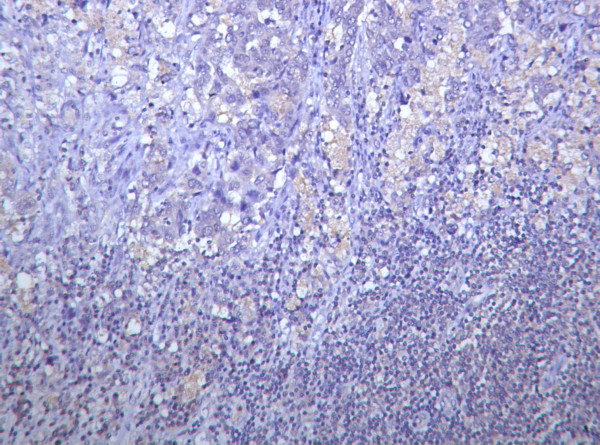
Photomicrograph showing absent nm23 expression in metastatic breast carcinoma. (Nm23 immunostain).

Nm23 immunoreactivity was absent to weak in metastatic tumour cells (Fig. 5). The score range was 0–6(mean = 1.240, SD = 1.850).

In this study there were 27 cases that showed spectrum of lesions ranging from normal to metastatic disease. There was a progressive down regulation of nm23 expression with neoplastic transformation; so that scores of 9 in non-proliferative breast disease decreased to 6 in carcinoma in-situ, to 3 in invasive cancer and 0 in meatstatic cancers.

## Discussion

Oncogenes (C-erb2, C-myc, genes linked to 11q13, tumour suppressor genes (retinoblastoma gene, p53) and anti-metastatic gene (nm23) play important roles in breast cancer progression [20]. Several investigators have reported the probable inverse association of nm23 expression with disease prognosis and or metastasis [21]. Metastatic process involves activation and down regulation of multiple genes at each step of metastatic cascade. Though there have been conflicting reports of increased nm23 expression in solid tumours (breast, colon, cervix, melanoma) compared to the benign counterparts [22,23]. Recent studies, however, are more consistent with that of nm23 functioning as a metastatic suppressor gene.

Royds et al [24] studied expression of nm23 in a variety of breast lesions. All of their benign categories showed uniform epithelial cell staining. In in-situ category non-comedo DCIS were positive whereas the comedo variants were negative for the protein expression. They also found that nm23 negativity was significantly associated with worsening grade of ductal carcinoma and advancing lymph node stage. They concluded that nm23 protein expression decreases with advancing grade of breast lesions and the negativity in comedo variant of DCIS is a finding consistent with the fact that comedo histology is known to have higher likelihood of becoming invasive.

Several other genetic alterations have been found to be consistently associated with such tumour progression apart from nm23. These include the FHIT gene located at human chromosome 3p14.2, novel genes called mta1(in rodents) or MTA1 (in humans), NES1, and the tumour suppressor gene maspin [25-29]

In the present study, our aim was to evaluate the modulation of nm23 expression in breast lesions as they progress from "normal" to "invasive cancer" through varying stages of "proliferative breast diseases". We detected the nm23 expression using immunohistochemistry and all the non-proliferative lesions strongly expressed the protein. Expression of nm23 was moderate in fibroadenoma, proliferative breast diseases and in carcinoma in-situ. In invasive carcinoma and metastatic diseases the nm23 expression was weak to absent. The difference in staining in various subgroups was also statistically significant. Thus our results show that there is down regulation of nm23 expression with the progress of neoplastic transformation. These results implicate that lack of nm23 expression in early lesions may be predictive of progression to invasive carcinoma. Though the findings in our study are in concordance with that of Royds et al [24], we found no difference in staining pattern between comedo versus non-comedo variants of DCIS. Also no difference in staining pattern between the morphologic variants of carcinoma (ductal/lobular) was detected in the present study.

Nm23 expression has been widely studied in various cancers and with their relation to staging and prognosis. There are very few studies in literature which have systematically studied the expression of this protein in both benign and malignant counterparts as spectrum. Our study is one such attempt and the results implicate that lack of nm23 expression in early lesions may be predictive of progression to invasive carcinoma and thus could be helpful in predicting the aggressiveness of the disease.

## Competing interests

The authors declare that they have no competing interests.

## Authors' contributions

ABa and KJo participated in selecting cases, carrying out immunohistochemistry, interpretation of results, and writing of the manuscript, RLo and BRa participated in the histopathological diagnosis, and editing of the manuscript, RSi provided the clinical details of the patients. All authors read and approved the final manuscript.
